# The Spatial Dynamics of Homelessness in Australia: Urbanisation, Intra-City Dynamics and Affordable Housing

**DOI:** 10.1007/s12061-022-09435-5

**Published:** 2022-01-29

**Authors:** Deb Batterham, Melek Cigdem-Bayram, Sharon Parkinson, Margaret Reynolds, Gavin Wood

**Affiliations:** 1 Centre for Urban Transitions, Swinburne University of Technology, Melbourne, Australia; 2 Launch Housing, Melbourne, Australia; 3 Centre for Just Places, Jesuit Social Services, Melbourne, Australia; 4 Social and Global Studies Centre, School of Global, Urban and Social Studies, RMIT University, Melbourne, Australia; 5 Centre for Social Impact, Swinburne University of Technology, Melbourne, Australia; 6 Centre for Urban Research, RMIT University, Melbourne, Australia; 7 School of Economic and Finance, Curtin Business School, Curtin University, Perth, Australia

**Keywords:** Homelessness, Urbanisation, Severe crowding, Couch surfing, Composition, Affordable housing

## Abstract

While homelessness in Australia has remained relatively stable at the national level, its spatial distribution is becoming more nuanced. This paper draws on homelessness estimates produced by the Australian Bureau of Statistics to explore the spatial dynamics of homelessness in Australia over a fifteen-year period. Building on existing work, we show that homelessness is becoming more urbanised with evidence of spatial convergence, mainly driven by a surge in severe crowding in our major cities. However, when exploring intra-city patterns, we find distinctive intra-city spatial dynamics featuring strong suburbanisation of ‘couch surfing’ in state capital cities, contrasting with shifts in severe crowding numbers toward middle and inner regions of most state capitals. We argue that these dynamics reflect the retreat of affordable rental housing supply to the outer suburban fringe, and the coping strategies that ‘couch surfers’ and those aspiring to live in the inner cities are compelled to follow in response to the changing spatial configuration of affordable housing.

## Introduction

Homelessness is a significant social problem in Australia. More than 116,000 Australians experienced homelessness on Census night in 2016, equivalent to 50 in every 10,000 people (ABS, 2018b). Many more experience homelessness and seek support from specialist homelessness services over the course of a year (AIHW, 2020). Like other social phenomena, homelessness is unevenly distributed across Australia. Mapping the spatial distribution of homelessness helps to guide the allocation of resources such as homelessness services and adds spatial nuance to a policy area that has, to date, been notably aspatial. Furthermore, identifying spatial variations in homelessness can aid our understanding of the relationship between homelessness and wider social and economic change in societies. Despite its importance, descriptive analysis of the geography of homelessness has been largely neglected, both in Australia and internationally.

Numerous studies have examined individual experiences of becoming, exiting and living through homelessness in specific places (e.g. Culhane et al., 1996; Jackson, 2012; Metraux et al., 2016; Rollinson, 2003). Others have sought to draw inferences about the causes of homelessness from spatial variation in homelessness rates and their relationship to area-based factors such as housing and labour market conditions, poverty rates, deinstitutionalisation and demographic patterns (e.g. Bohanon, 1991; Elliott & Krivo, 1991; Honig & Filer, 1993; Lee et al., 2003; Quigley et al., 2001). A few homelessness researchers have worked to quantify the geography of homelessness in the U.S. and U.K. (e.g. Bramley et al., 2015; Lee & Price-Spratlen, 2004). In Australia, Chamberlain and MacKenzie (2002, 2008) observed substantial variation in homelessness rates between states and territories, while Wood et al., (2014) documented variation in homelessness rates using finer spatial units. More recently, Pawson et al. (2018, 2020) analysed the geography of Australian homelessness in recent years to monitor broader service system needs and practices, while O’Donnell (2016) explored how different types of homelessness in Sydney are closely associated with locational characteristics. He found that boarding houses and homelessness services (homelessness in congregate settings) are typically found in inner city locations, as are ‘rough sleepers’ (people sleeping on the street, in cars, squat or improvised dwellings), who commonly access these forms of assistance. In contrast, severe crowding and other forms of homelessness associated with private accommodation have a more suburban orientation.

Focusing on the 2001–2016 Censuses, Parkinson et al., (2019) analysed Australia-wide changes in the geography of homelessness and found that strong growth in severe crowding homelessness has accompanied the growing urban concentration of homelessness in state capital cities. Building on this work, this paper documents shifts in the intra-city geography of homelessness and explores the role played by the changing spatial pattern of affordable housing supply in prompting shifts in the location of severe crowding and ‘couch surfing’ (staying temporarily with other households) forms of homelessness.

The paper proceeds as follows. In section 2, data sources and definitions of homelessness are explained. Section 3 follows with descriptive analyses of how Australia’s spatial dynamics of homelessness unfolded over the 2001–2016 study timeframe. Section 4 outlines how these dynamics are accompanied by an urbanisation of homelessness and investigates changing intra-city patterns in homelessness, particularly focusing on severe crowding and couch surfing while section 5 explores the role of housing affordability in these shifting intra-city patterns. A concluding section summarises, draws some implications for Australian policy, and outlines future directions for research.

## Data, Homeless Definitions and Spatial Units

This study relies on the homelessness estimates generated by the Australian Bureau of Statistics (ABS) from the five-yearly, Census of Population and Housing. The estimates are derived using an enumeration strategy applied to the Census data since 1996, producing a nationwide, point-in-time homelessness count (ABS, 2018b; Chamberlain & MacKenzie, 2002). The ABS has published these estimates on a consistent methodological basis from each of the 2001, 2006, 2011 and 2016 Censuses.^1^ The ABS enumerate those experiencing homelessness based on personal circumstances observed on Census night and assumptions about the way people respond to Census questions if homeless (ABS, 2012b, 2018b).^2^

We use Census data because it provides the best available time series measure of the geography of homelessness, encompassing a holistic definition of its multiple dimensions.^3^ The ABS statistical definition of homelessness emphasises the ‘home’ in homelessness, where ‘home’ encompasses a sense of security, stability, privacy, safety and the ability to control one’s living space (ABS, 2012a; Mallett, 2004). Homelessness within this definition is a loss of one or more of these elements and not just about ‘rooflessness’. Therefore, someone is defined as homeless if they do not have suitable alternative accommodation and their current living arrangement is in an inadequate dwelling, or has no tenure, or their tenure is short and cannot be extended, or does not allow them to have control of, and access to space for social relations (ABS, 2012a).

The ABS homelessness estimates classify those experiencing homelessness into six operational groups. Table 1 lists homeless counts and rates of homelessness (per 10,000 persons) for each group and Census year. These groupings represent different types of homelessness that the empirical analysis uses as a lens through which to view the changing geography of homelessness, and thereby gain better understanding of its meaning and implications. Table 1 shows that severe crowding has soared since 2006 to become the dominant type of homelessness.

**Table 1:** National counts and rates (per 10,000 persons): ABS homeless operational groups, 2001–2016

	2001		2006		2011		2016	
	Count	Rate	Count	Rate	Count	Rate	Count	Rate
Persons living in improvised dwellings, tents, or sleeping out	8946	4.8	7247	3.7	6810	3.2	8200	3.5
Persons in supported accommodation for the homeless	13,420	7.2	17,329	8.7	21,258	9.9	21,235	9.1
Persons staying temporarily with other households (couch surfers)	17,880	9.5	17,663	8.9	17,374	8.1	17,725	7.6
Persons living in boarding houses	21,300	11.4	15,460	7.8	14,944	6.9	17,503	7.5
Persons in other temporary lodgings	338	0.2	500	0.3	682	0.3	678	0.3
Persons living in severely crowded dwellings^a^	33,430	17.8	31,531	15.9	41,370	19.2	51,088	21.8
All homeless persons	**95,314**	**50.8**	**89,728**	**45.2**	**102,439**	**47.6**	**116,427**	**49.8**

Source: ABS (2018a), Table 1.1 Homeless Persons, Selected characteristics, 2001, 2006, 2011 and 2016

^a^Severe crowding is defined as occurring when four or more extra bedrooms are needed to appropriately accommodate the residents of a dwelling. This threshold is based on the Canadian National Occupancy Standard, which specifies that no more than two persons should share a room with specific clauses about the age, gender and relationship between the occupants

Statistical Area Level 3 (SA3) is the smallest ABS-defined spatial unit for which operational group estimates are available. There are 334 SA3s in 2016 Australia-wide with populations over 100. These SA3s have an average 2016 population of 70,000 and range from roughly 10,000 to 230,000 people. Intercensal changes in spatial unit boundaries were addressed using ABS population weighted correspondence files that apportion data to the 2016 SA3 boundaries.

**Fig. 1:**
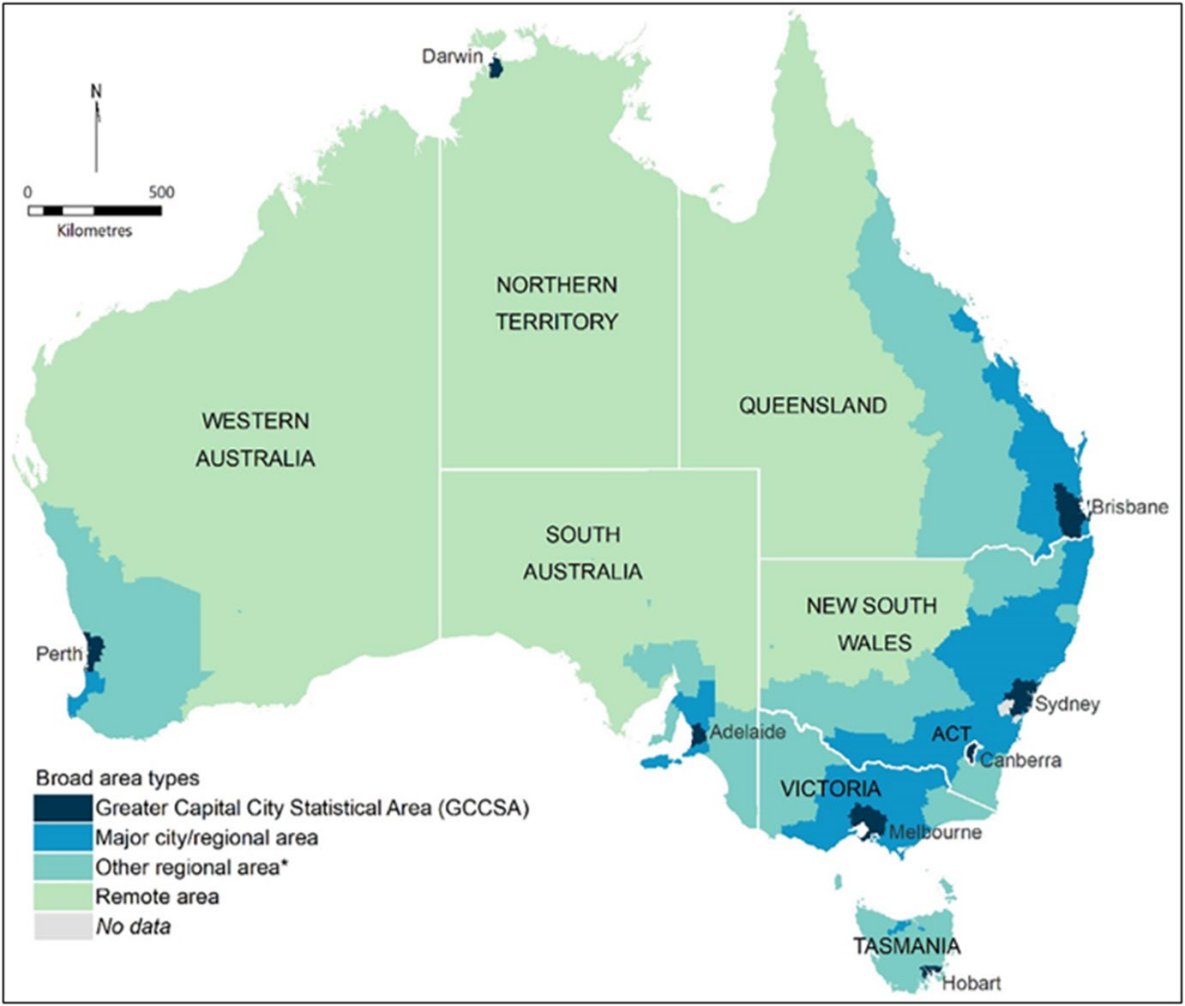
Four broad area types used in analysis

Descriptive analyses of the spatial dynamics of homelessness draws upon a modified version of the ABS ‘Remoteness Area’ (RA) classification – a system that divides Australia into five area-types based on road access to services.^4^ The ABS RA categories were adjusted as follows: greater capital city areas were defined for each state and territory using an aggregation of SA3s within the ABS Greater Capital City Statistical Area (GCCSA) boundaries; other ‘Major Cities’ outside the capitals were combined with ‘Inner Regional’ areas; and ‘Remote’ and ‘Very Remote’ areas were combined. As a result, the 334 SA3s were classified into the following four broad area types (mapped in Fig. 1):

- Greater Capital City Statistical Areas (GCCSA) (185 SA3s; 66% of population)
- Major city and regional areas (89 SA3s; 24% of population)
- Other regional areas (44 SA3s; 8% of population)
- Remote areas (16 SA3s; 2% of population)

Shifts in the geography of homelessness across these broad area groupings are reported in Sections 3 and 4.

**Fig. 2:**
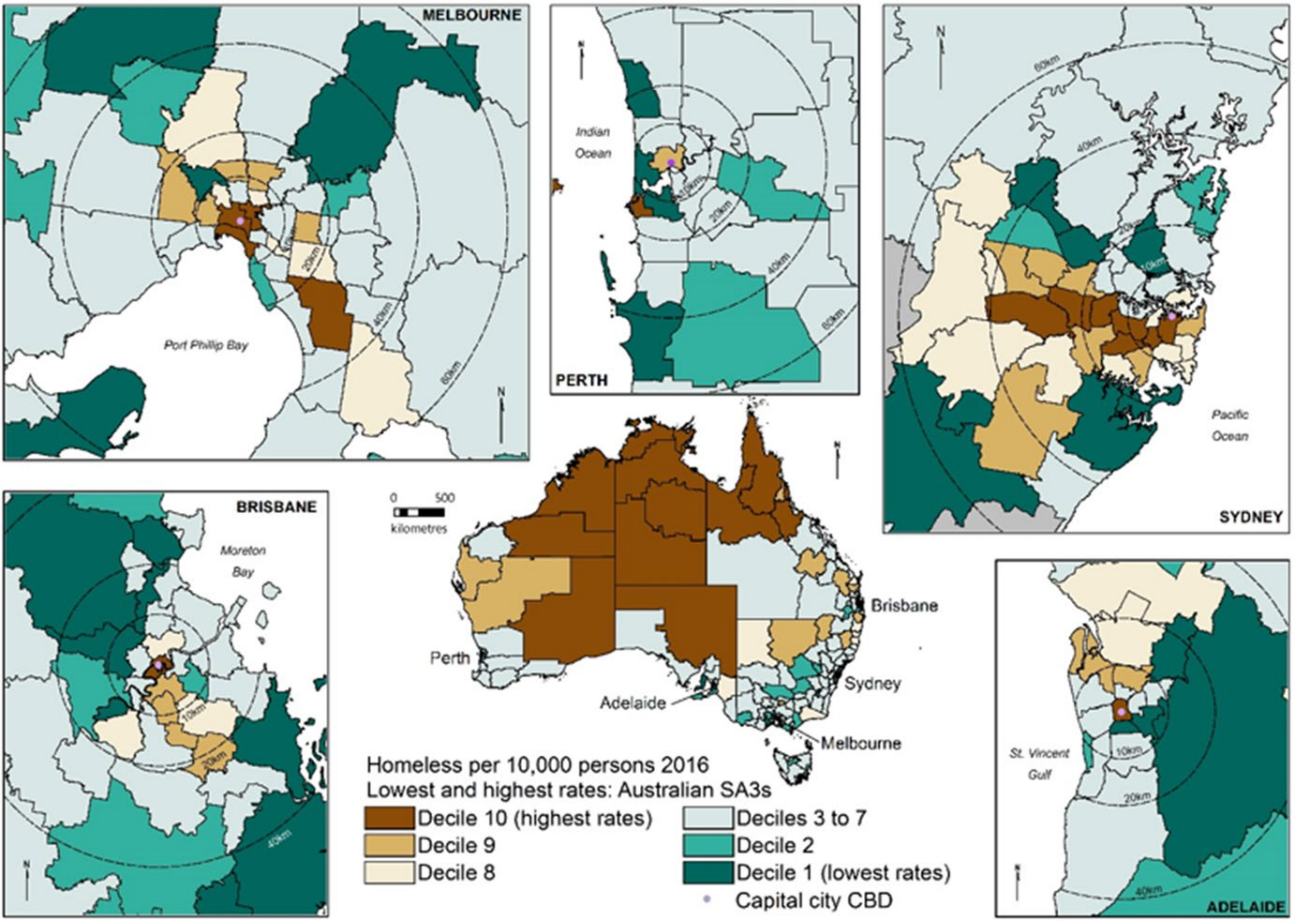
Lowest and highest rates of homelessness (by decile), selected state capital cities and Australian SA3s, 2016

**Fig. 3:**
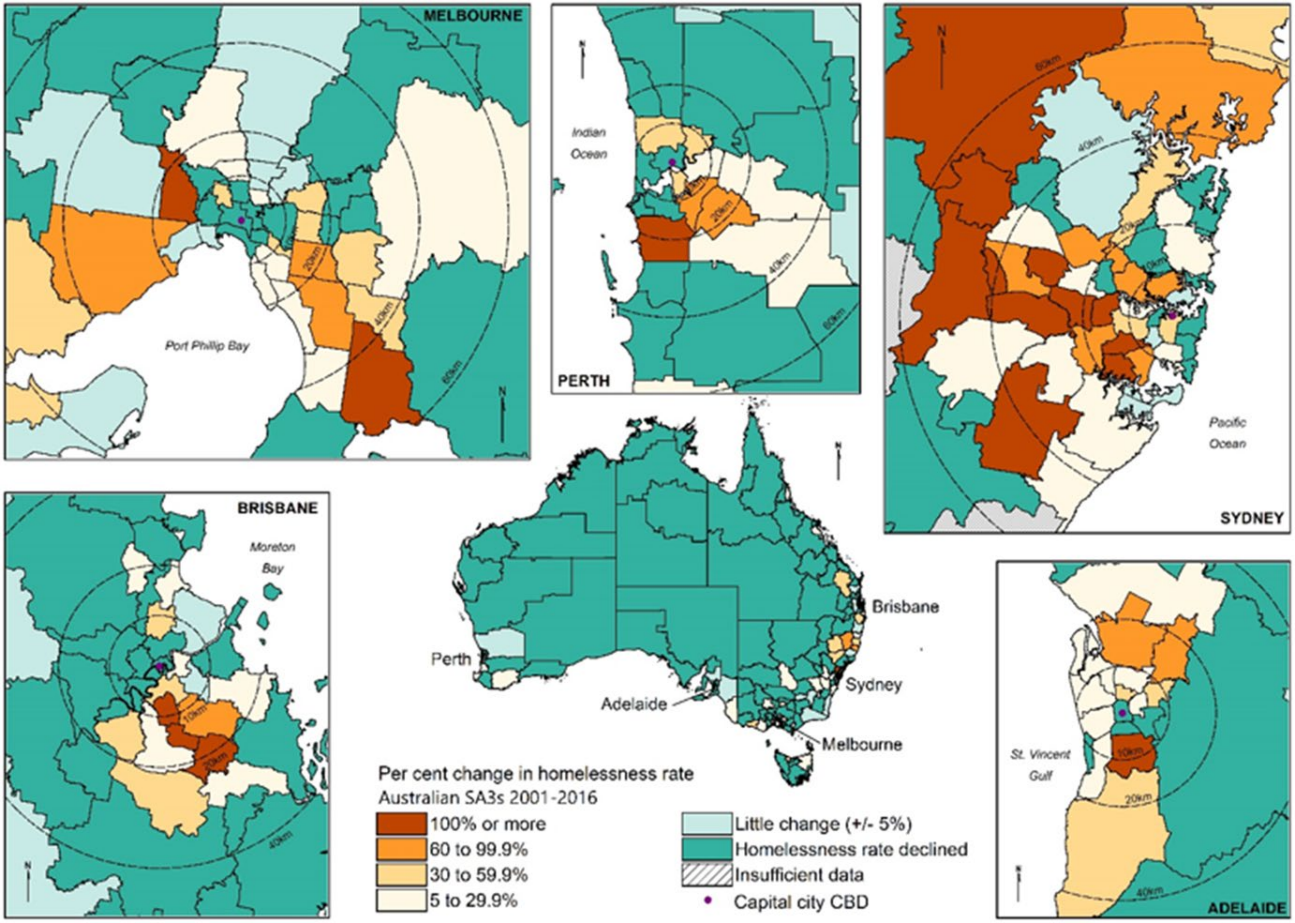
Percentage change in homelessness rates, selected state capital cities and Australian SA3s, 2001 to 2016

Intra-city changes in the distribution of homelessness are analysed by dividing the five most populous state capital cities into inner (SA3s within approximately 10 km of the CBD), middle (SA3s 10-20 km from the CBD) and outer ring suburbs (SA3s 20 km or more from the CBD), with distances measured from the CBD point to the centroid of a given SA3. CBD distance rings are shown in Figs. 2 and 3.^5^

Customised ABS Census data was obtained to determine the supply of private rental housing affordable to those on low incomes, specifically, by SA3: a count of private rental sector (PRS) households in the bottom 40% of the national household income distribution; and a count of PRS dwellings affordable^6^ to this group. A shortage (or surplus) of affordable PRS dwellings was calculated for each SA3 by simply subtracting the number of lower income PRS households from the number of affordable dwellings in each SA3.

## The Changing Spatial Dynamics of Homelessness in Australia, 2001–2016

Figure 2 maps the SA3 regions grouped into deciles based on their 2016 homelessness rate. Substantial variation is evident with a median rate in the highest decile thirteen times the median rate in the lowest. The highest rates of homelessness are found in remote and very remote areas of central, northern and western Australia as well as in Central Business Districts (CBDs) and adjacent areas of the larger capital cities.

Source: Parkinson et al., (2019), Fig. 3, p 24. Figure 2. uses ABS Census and Census Homelessness Estimates 2016; ABS digital SA3 Statistical Geography Boundaries, 2016.

Figure 3 presents changes in homelessness rates (per 10,000 persons) between 2001 and 2016 and shows clearly discernible spatial patterns. In much of regional and remote Australia homelessness rates declined. Conversely, much of metropolitan Australia has witnessed rising homelessness. Rising homeless rates are by no means an inner-city phenomena: there are clear indications in the capital city inserts in Fig. 3, of a surge in homeless rates in many suburban metropolitan areas though often from relatively low initial rates.

**Fig. 4:**
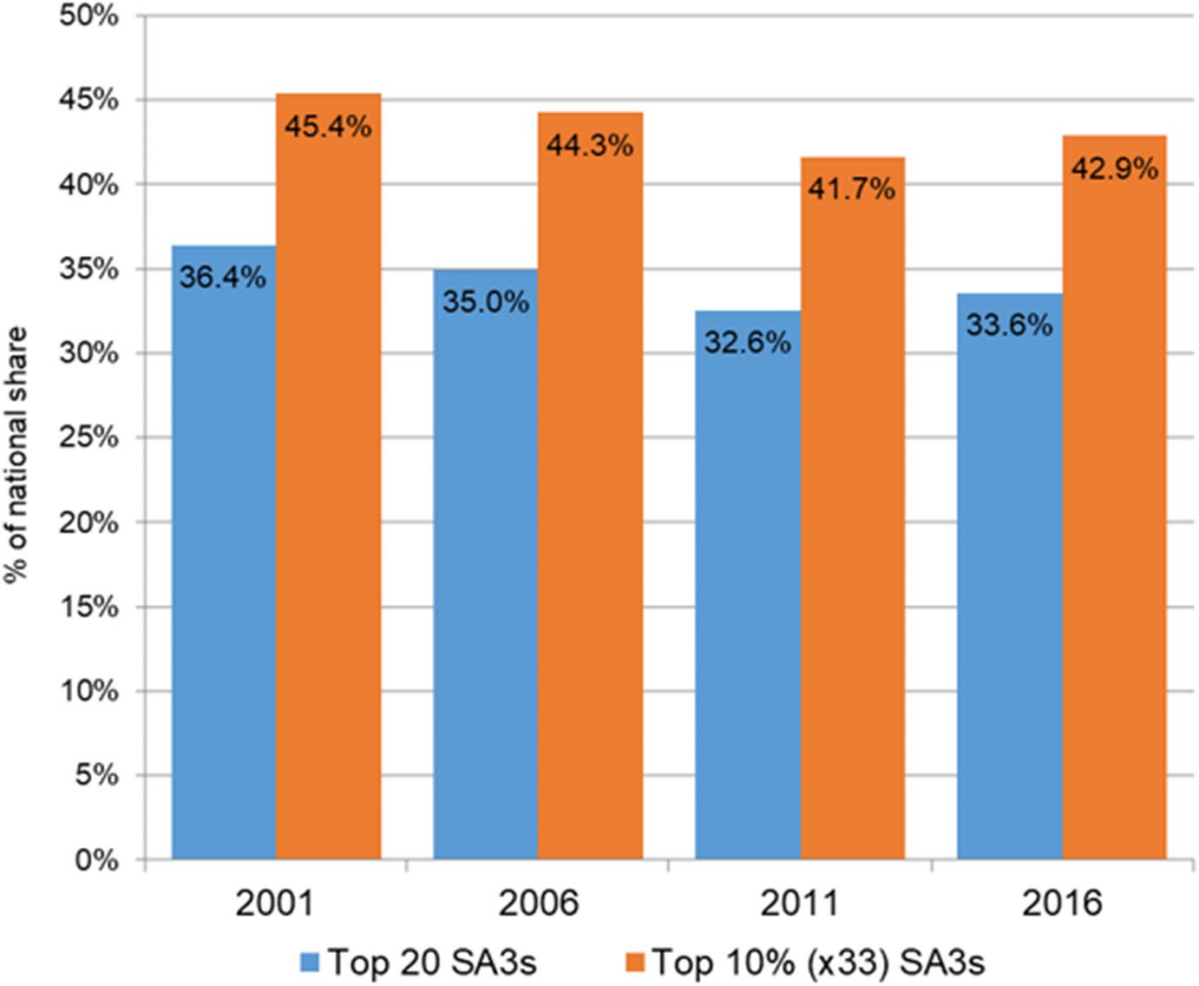
Concentration ratios, share of national homelessness accounted for by the top 20 and top 33 (10%) SA3s, 2001–2016

These changing spatial patterns can be quantified using measures of spatial concentration that gauge whether homelessness is becoming more or less prominent in those regions with the highest homeless counts. For example, the n% concentration ratio is the percentage of the national homeless count located in the n% SA3s with the highest homeless counts. Figure 4 reports estimates for 2001 through to 2016 and for top 33 (10%) and top 20 (≈ 5%) SA3 ratios. In 2001, a little over one-third (36%) of the national homeless count was found in the top 5% regions, but there was a trend decline through to 2011. There is a partial reversal in the final 2011–2016 intercensal period, a pattern that is repeated using the 10% concentration ratio.^7^

**Fig. 5:**
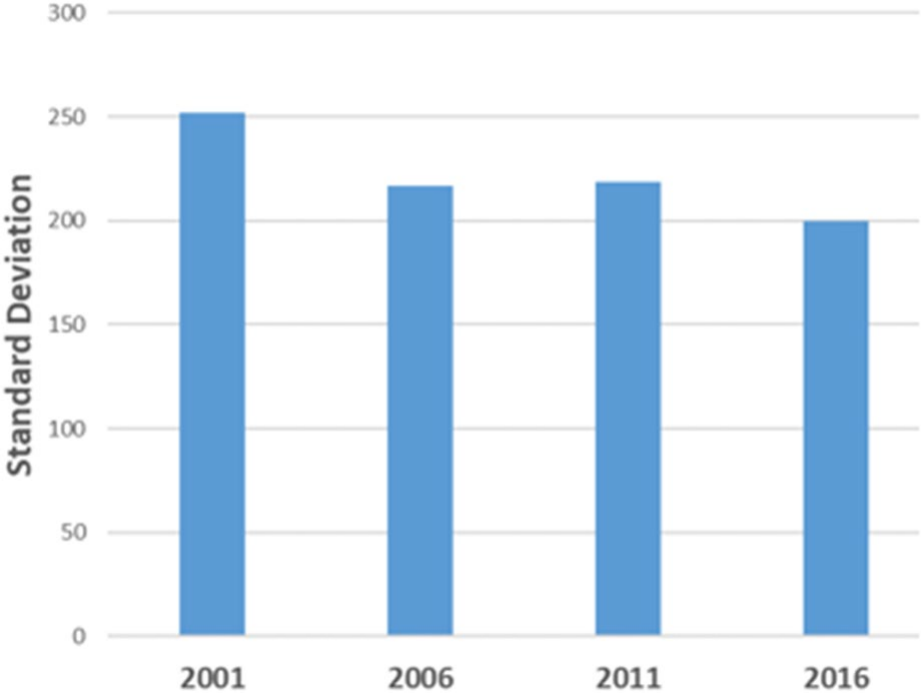
Standard deviation of rates of homelessness 2001–2016

**Fig. 6:**
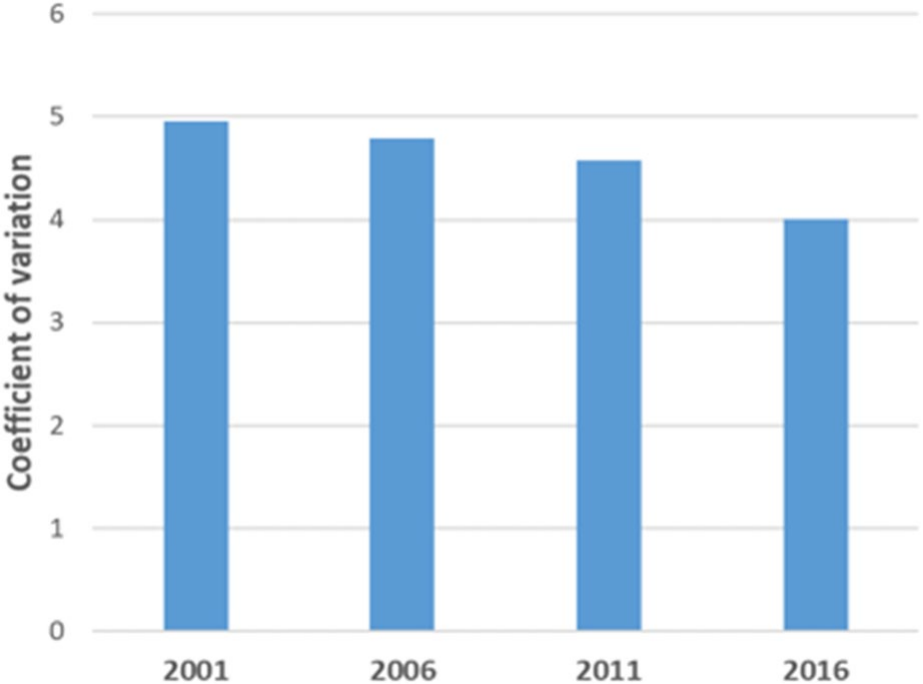
Coefficient of variation of rates of homelessness 2001–2016

Figures 5 and 6 report standard deviations of regional per capita homeless rates and coefficients of variation to determine the degree of dispersion in homelessness rates over the data range. Figure 5 documents a trend decline in standard deviation of homeless rates (per 10,000) falling by 20% between 2001 and 2016. A coefficient of variation which normalises the standard deviation using the mean regional rate confirms the decline (see Fig. 6). These results suggest a convergence in rates of homelessness as a more even spatial distribution of homelessness emerges over the time period. The next section explores what these spatial dynamics mean for the geography of homelessness in Australia.

## Urbanisation and the Intra-City Geography of Homelessness

In this section, two key trends underpinning the spatial dynamics of homelessness are explored. First, we document how homelessness has become more urbanised through a high rate of growth in capital cities relative to regional and remote areas. Second, we show how the intra-capital city geography of different types of homelessness is shifting in divergent ways.

### Urbanisation of Homelessness

**Fig. 7:**
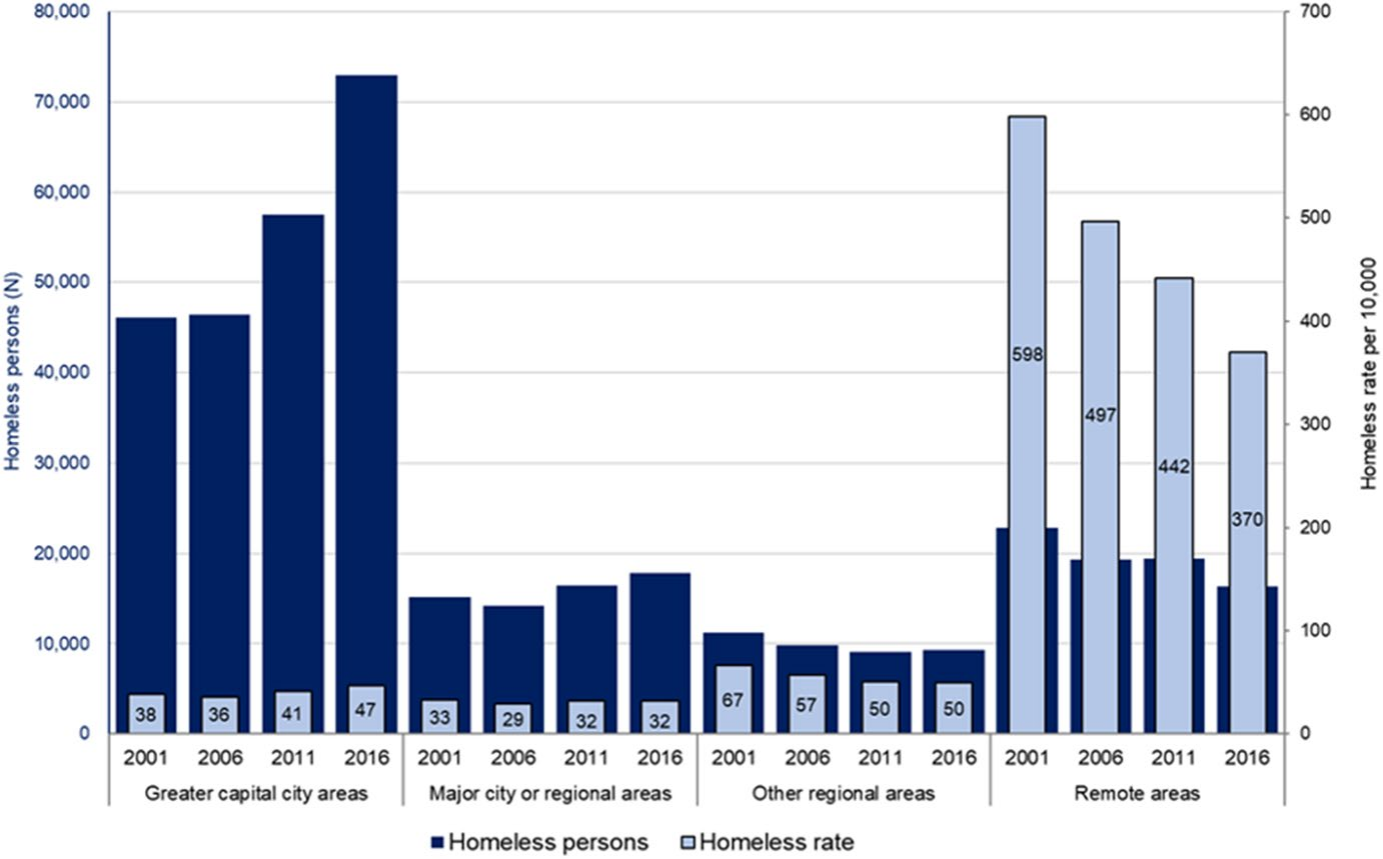
Number and rate of homeless by area type: 2001, 2006, 2011 and 2016

Homelessness rates have diverged from the national population’s dynamics as they play out between capital cities and the rest of Australia. Figure 7 shows how homeless counts and rates of homelessness have evolved across greater capital cities, major cities and regions, other regional areas and remote areas between 2001 and 2016. It documents an increase in the capital city rate of homelessness from 38 to 47 per 10,000 population, which translates into a large 26,899 (58%) increase in the capital city homeless count (from 46,059 to 72,958). Meanwhile, the rate plummets in remote areas, from 598 to a still high 370, while the remote homeless count falls from 22,796 to 16,300.

**Fig. 8:**
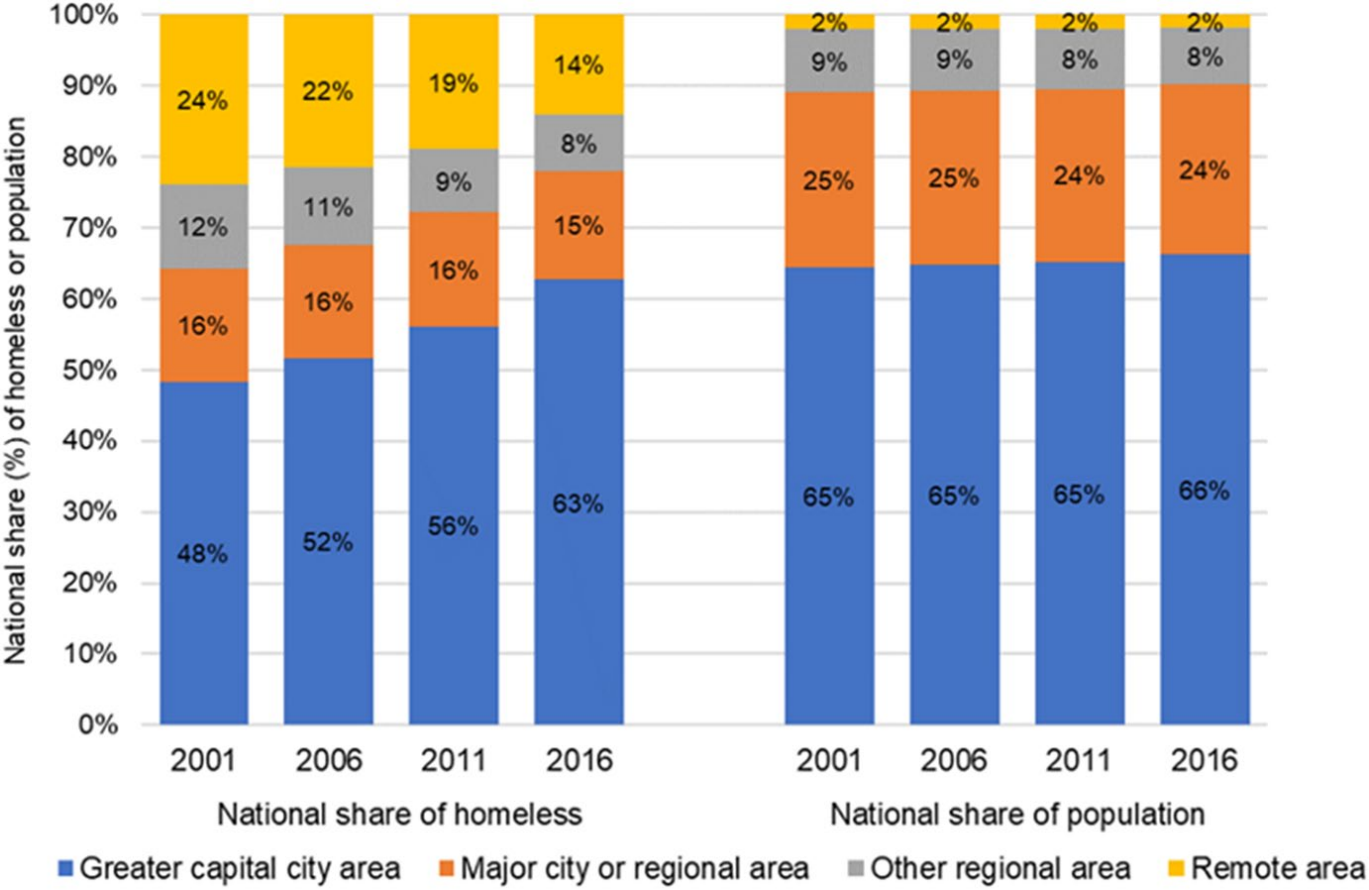
National shares (%) of homeless persons and population by area type: 2001, 2006, 2011 and 2016

Figure 8 documents each area type’s share of the national homeless count. The capital cities share of the national count soars, from a little under one-half (48%) in 2001, to nearly two thirds (63%) in 2016. Yet the share of the national population in capital cities was roughly unchanged over the study period: the rise in the capital cities share of total homelessness is not, therefore, replicating a corresponding increase in the capital city share of the national population.

Table 2 drills down to describe the changing geography of particular types of homelessness to shed some light on what lies behind its urbanisation. Over 2001–2016, there are increases in capital cities per capita supported accommodation (+34.5%) and to a much lesser extent rough sleepers (+3.2%, but from low base). These increases offset falls in boarding house (−28.4%) and ‘couch surfing’ (−26.0%) rates of homelessness. But the overwhelming momentum behind the surge in capital city homeless rates is severe crowding. Its per capita rate in capital cities almost triples, from 7.4 to 20.0. Severe crowding becomes the dominant presence in capital cities’ homeless profiles with 2016 rates exceeding those of all other types of homelessness. The nearly threefold increase contrasts with the marked decrease in severe crowding outside capital cities. Consequently, the national distribution of those living in severely crowded dwellings is transformed: in 2001, 27% were in capital cities but by 2016 this share increased to 60%. These patterns presumably reflect changed conditions within our largest cities that are absent or weaker elsewhere.

**Table 2:** Rates per 10,000 persons for each homelessness operational group, by greater capital city and balance of state area, 2001–2016

	2001	2006	2011	2016	% change 01 to 16
**1 Persons who are in improvised dwellings, tents or sleepers out (rough sleepers)**					
Greater capital city	2.4	2.1	2.0	2.5	3.2
Outside capital city	9.1	6.5	5.3	5.5	−39.4
**Total**	**4.8**	**3.7**	**3.1**	**3.5**	**−26.7**
**2 Persons in supported accommodation for the homeless**					
Greater capital city	7.0	9.0	9.8	9.5	34.5
Outside capital city	7.4	7.4	10.1	8.3	12.8
**Total**	**7.2**	**8.4**	**9.9**	**9.1**	**26.8**
**3 Persons staying temporarily with other households (couch surfers)**					
Greater capital city	8.3	7.8	7.0	6.2	−26.0
Outside capital city	10.9	10.9	10.1	10.4	−4.6
**Total**	**9.3**	**8.9**	**8.1**	**7.6**	**−17.8**
**4 Persons staying in boarding houses**					
Greater capital city	12.5	8.9	8.5	9.0	−28.4
Outside capital city	9.0	5.7	4.1	4.6	−48.8
**Total**	**11.3**	**7.7**	**7.0**	**7.5**	**−33.5**
**5 Persons in other temporary lodging**					
Greater capital city	0.1	0.2	0.2	0.1	26.2
Outside capital city	0.3	0.4	0.4	0.5	74.9
**Total**	**0.2**	**0.3**	**0.3**	**0.3**	**51.0**
**6 Persons living in ‘severely’ crowded dwellings**					
Greater capital city	7.4	8.0	13.4	20.0	170.7
Outside capital city	36.8	30.4	30.3	25.5	−30.6
**Total**	**17.8**	**15.9**	**19.3**	**21.9**	**22.6**

Note: The ABS suppress the count for operational groups in SA3s that record very low numbers. Table 2 is derived from aggregating larger SA4 spatial units, for which suppression is minimised. In some suppressed SA4s, data for some internal SA3s was available, and these counts have been added to city or balance totals. In 2001, data for operational group 2 was not available below the state level and thus these counts have been imputed for city and balance areas using the strategy outlined in Wood et al., (2014). Consequent of these data issues, the numbers do not exactly match the figures reported in Table 1, as the latter are sourced directly from ABS published, national level, unsuppressed data

### Shifting Intra-City Location

O’Donnell (2016) also acknowledges evidence of a more even spatial distribution of Australian homelessness. He links this development to changing spatial patterns of different forms of homelessness in Sydney. Severe crowding is found to significantly alter Sydney’s geography of homelessness because of its association with suburban private dwellings, and its strong growth. Severe crowding’s spatial pattern contrasts with that of homelessness associated with non-private dwellings (boarding houses and supported accommodation), and of rough sleepers, which remains concentrated in and around inner city Sydney locations.

**Fig. 9:**
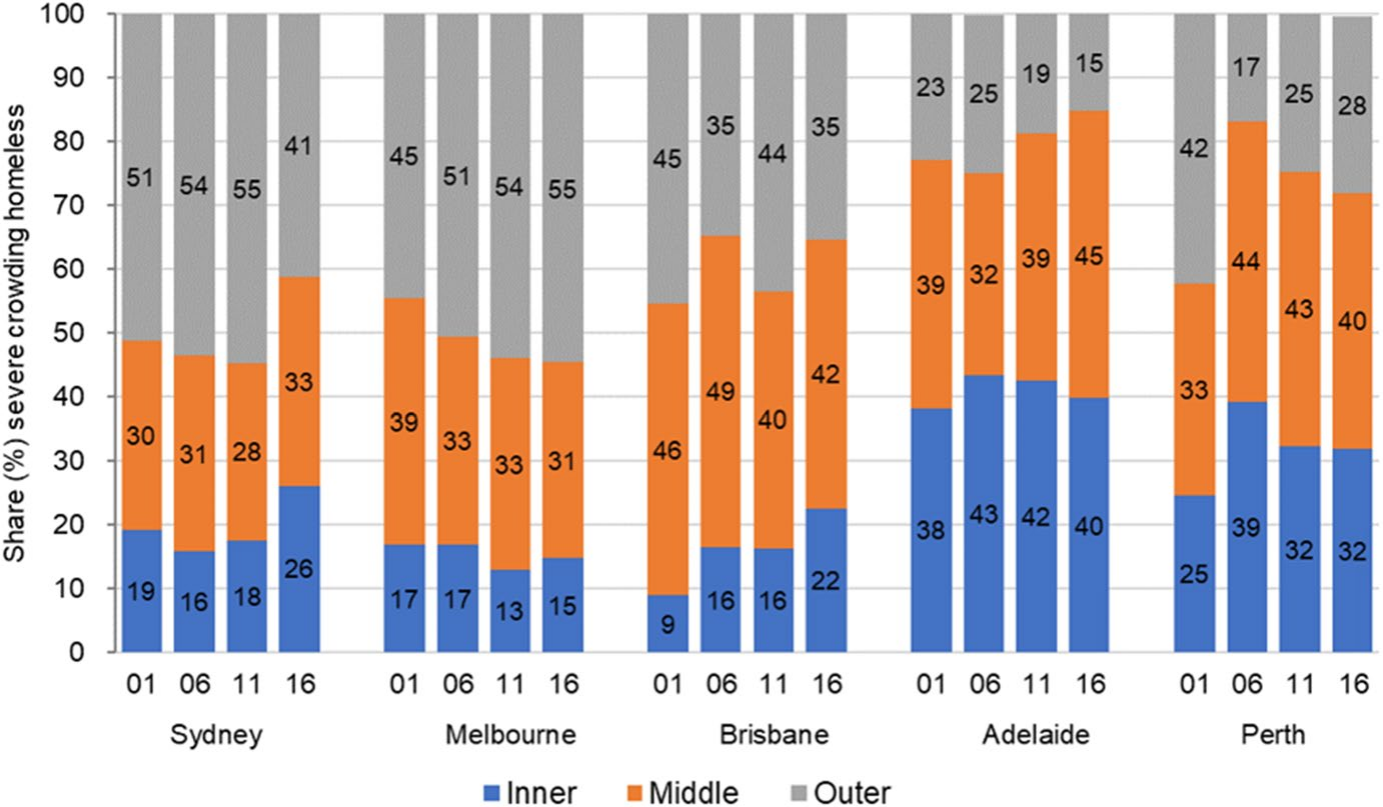
Intra-city spatial patterns 2001–2016: Severe crowding

Figure 9 illustrates the spatial shifts in shares of severe crowding in the inner, middle, and outer areas of Australia’s five largest capital cities. With the exception of Melbourne, outer suburban shares of overcrowding decline, and sharply so in Brisbane and Perth, as the geographical distribution shifts toward middle and inner suburbs; this change is especially pronounced in Brisbane, where the inner suburbs share of severe crowding more than doubles (9% to 22%). Sydney and Perth inner area shares of severe crowding both increase by 7 percentage points, while inner Adelaide’s severe crowding share ends up marginally ahead of its 2001 share. Severe crowding’s stronger presence in middle, and especially inner suburbs, brings its spatial pattern closer to that of other forms of homelessness that began the observation period concentrated in these same inner suburbs.^8^

**Fig. 10:**
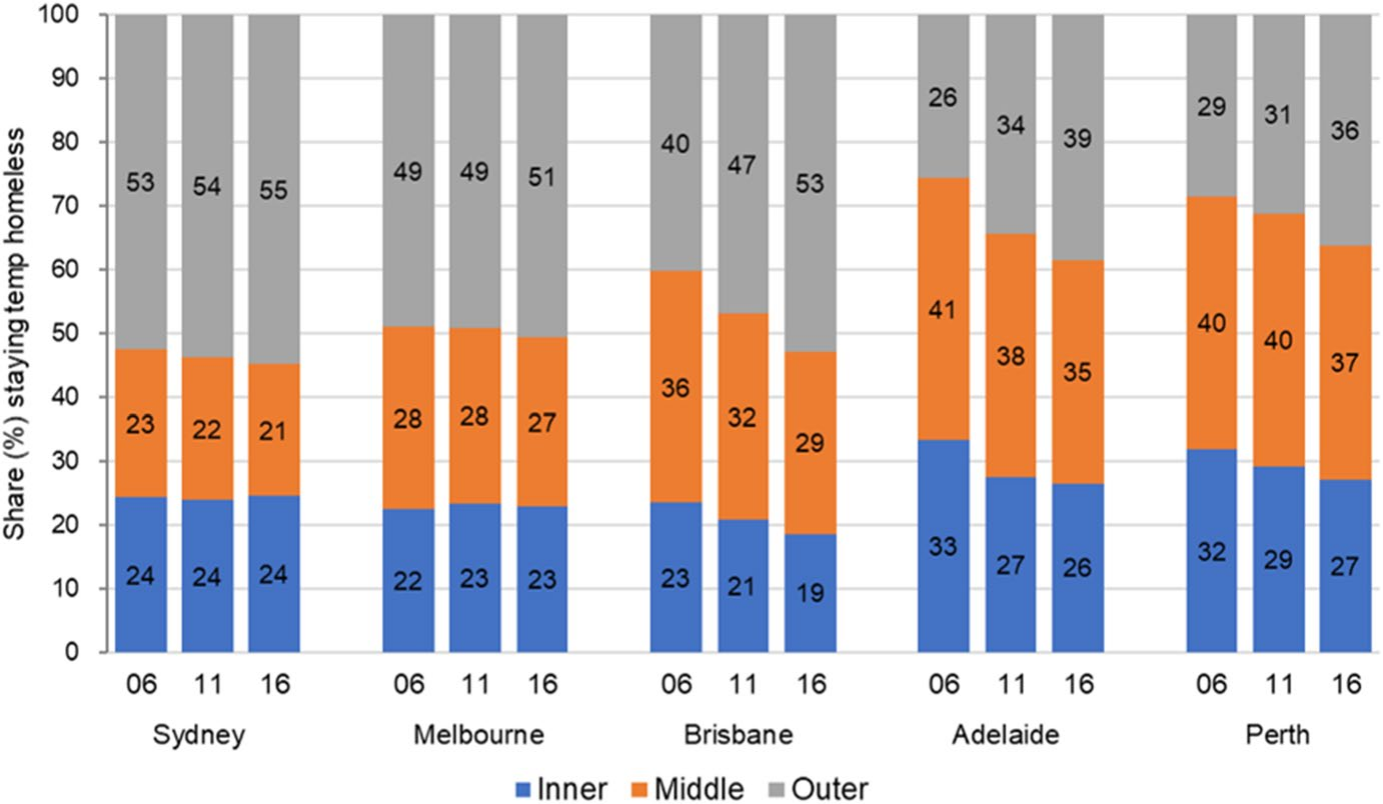
Intra-city spatial patterns: ‘Couch surfers’

However, there are contrasting dynamics with respect to ‘couch surfers’, the second most important homeless group (at the beginning of the study period). On drilling down to inspect their shifting spatial distribution, we discover strong suburbanisation to outer areas in all cities. This is consistent with Randolph and Tice's (2016) work on the suburbanisation of disadvatage in Sydney.. Suburbanisation is marked in Adelaide, Brisbane and Perth (Fig. 10), while in Melbourne and Sydney, the outer suburban share of ‘couch surfers’ edges up by a couple of percentage points. On the other hand, congregate homeless accommodation (boarding houses and supported accommodation) typically remains concentrated in inner and middle ring suburbs of these cities (not shown). Their continued inner-city concentration is perhaps linked with the persistent inner-city concentration of rough sleepers. Well over 50% of rough sleepers locate in the inner areas of these state capitals, a cut of all rough sleepers that is rising in Sydney, Melbourne, Brisbane and Adelaide, while enduring at very high shares in Perth.^9^

Melbourne is distinctive and curious because a drift to suburban locations is uniform across all types of homelessness other than rough sleepers. It is the only city where both the private housing that offers precarious or severely crowded accommodation, as well as congregate styles of accommodation, is increasingly found in outer suburban locations. This curious exceptionalism is not limited to one spatial dimension of Melbourne’s housing market. Phelps et al. (2021) find that Melbourne is the only state capital city with a spatial distribution of house prices that is diverging rather than converging, while Melser et al. (in review) report some evidence of a price elastic housing supply in Melbourne that contrasts with price inelastic supply in the other capital cities (as well as regional Australia).

## Housing Affordability and Shifting Intra-City Patterns

We now explore the relationship between the changing geography of affordable private rental housing stock and the shifting intra-city patterns of severe crowding and couch surfing homelessness. We hypothesise that shrinking stocks of affordable rental housing have shifted to the outer suburbs or capital cities, and as a result, those experiencing homelessness are compelled to adopt coping strategies that are reflected in a changing spatial configuration.^10^ These coping strategies differ across distinct subgroups among the homelessness population.

Rental housing affordability pressures have worsened in Australia over a period featuring strong national population growth, two thirds of which has been in Australia’s capital cities. Hulse et al. (2019) draws on 1996–2016 Australian Censuses to document growth in the supply of private rental housing in the mid-market range, but decline at the low, affordable end. They also find an increase in moderate to higher income renters occupying dwellings at the low and mid-ranges of the market, further exacerbating shortages of rental dwellings affordable to those with the lowest incomes.

We draw on Hulse et al.’s (2019) measure of the stock of private rental housing affordable to low income households to chart its changing spatial distribution in selected capital cities.^11^ Table 3 reports the results from Ordinary Least Squares (OLS) regressions of this affordable housing measure on distance from the CBD, as well as a population density variable.^12^ The higher are population densities, the greater are market pressures all else equal. While we can expect the distance from CBD and density measures to be correlated,^13^ a polynucleated structure in large capital cities means that rents and prices will have peaks around sub-metropolitan centres that the population density measure could capture. Table 3, panel A, reports coefficient estimates from a model estimated on 2001 data: two regressions are reported for each capital, one with and the other omitting population density. Panel B reports estimates from the same models but estimated using 2016 data.

**Table 3:** OLS regression estimates: dependent variable affordable rental housing stock

	Adelaide	Brisbane	Perth	Melbourne	Sydney					
	Model 1	Model 2	Model 1	Model 2	Model 1	Model 2	Model 1	Model 2	Model 1	Model 2
(a) In 2001										
Dist. from CBD	51.95	83.06	−21.71	5.856	−215.6	90.17	0.893	12.38	24.53***	20.93**
	(48.79)	(65.62)	(32.61)	(35.43)	(158.7)	(174.8)	(8.370)	(15.58)	(5.775)	(9.552)
Pop. Dens. ‘01	N/A	0.161	N/A	0.254*	N/A	1.118**	N/A	0.197	N/A	−0.0612
		(0.223)		(0.145)		(0.400)		(0.225)		(0.129)
Constant	776.1**	367.8	1277***	836.3**	2846**	68.61	1312***	746.6	−642.0***	−436.4
	(352.4)	(668.9)	(283.3)	(373.4)	(1033)	(1333)	(237.9)	(689.2)	(194.5)	(474.8)
Observations	19	39	21	40	46					
R-squared	0.063	0.092	0.012	0.089	0.088	0.364	0.0003	0.021	0.291	0.294
(b) 2016										
Dist. from CBD	185.6***	182.3***	99.02***	80.56**	141.6**	210.6***	24.56**	−20.08	26.04***	−3.500
	(44.69)	(57.32)	(28.46)	(30.68)	(56.93)	(70.22)	(11.40)	(19.50)	(6.529)	(8.444)
Pop. Dens. ‘16	N/A	−0.0171	N/A	−0.135	N/A	0.218	N/A	−0.601***	N/A	−0.399***
		(0.179)		(0.0913)		(0.139)		(0.221)		(0.0874)
Constant	−466.1	−420.1	−992.6***	−691.7**	−1296***	−1936***	−326.1	1843**	−2082***	−405.9
	(322.8)	(584.6)	(247.3)	(317.4)	(370.6)	(541.5)	(324.1)	(851.5)	(219.9)	(410.1)
Observations	19	39	21	40	46					
R-squared	0.5035	0.5038	0.247	0.290	0.246	0.337	0.109	0.257	0.266	0.505

Notes: Standard errors in parentheses, *** p < 0.01, ** p < 0.05, * p < 0.1; Authors’ own calculations using ABS customised Census data

The estimated constant in 2001 regressions omitting population density is invariably positive, and implies that cities’ stocks of affordable housing are sufficient to accommodate low-income households in the CBD. Moreover, there is no significant relationship between affordable stock and distance from the CBD while each regression’s R^2^ is very low (Adelaide, Brisbane and Melbourne) or low (Perth and Sydney). In Sydney, however, there is weak evidence of a CBD shortage, and stronger evidence of a positive gradient in the distance from CBD and affordable rental housing relationship.^14^ On allowing for peaks and troughs in affordable housing stock correlated with peaks and troughs in population density, there is typically little improvement in R^2^s, and distance from CBD coefficients remain statistically insignificant. In Perth, however, there is a large improvement in the regression ‘fit’, and a positive and significant (at 5%) population density coefficient, suggesting the presence of sub-metropolitan centres with concentrations of affordable private rental housing.

With panel B’s 2016 estimates, a conspicuously different pattern of results emerges. Supplies of affordable housing retreat to the middle and outer suburbs of all capital cities.^15^ In regressions omitting population densities, we now find deficits in affordable private rental housing stocks in all cities’ CBDs, as evidenced by negative constant estimates. By 2016, a positive, statistically significant (at 5% or better) affordable housing - distance from CBD gradient has emerged in each capital city. A more accurate *description* of the geography of affordable housing emerges as the R^2^ in these regressions is (in all but one city) much higher than the R^2^ in equivalent 2001 regressions. On adding population density variables, these positive affordable housing – distance from CBD gradients persist in all cities other than the larger Melbourne and Sydney urban conurbations. In these cities, there is a marked polynucleated urban structure with sub-metropolitan centres such as Parramatta in Sydney, and Dandenong in Melbourne. The negative and significant population density coefficients in these cities’ regressions indicate that shortages in affordable rental housing are developing in these centres that are at some distance from the CBD.^16^

Some idea of how the two largest (in 2001) homelessness groups – severe crowding homeless and couch surfers – might have responded to these major shifts in the intra-city geography of affordable rental housing can be gleaned from Tables 4 and 5. They compare the 2016 socioeconomic and demographic profiles of severely crowded homeless and couch surfers Australia-wide, and in the largest state capitals. There are stark differences: the severely crowded homeless in Table 4 are drawn from younger age groups (nearly three quarters are under 35 years Australia wide); disproportionately born overseas, with the Asian born especially prominent^17^; and are in the early stages of household formation careers with a large majority either single, never married or married (91%). The Asian bias in demographic profiles is a recent development^18^ and more pronounced in Sydney and Melbourne, but otherwise personal characteristics are more or less uniform across state capitals. Being young, this group are drawn to inner suburbs, including areas surrounding major service economy hubs, where affordable rental housing has become scarcer over the study time frame (see Table 3). Relatively high housing costs compel low income younger persons (over half have incomes under $400 per week) to economise on the consumption of housing space, the product of which is group households and multigenerational households that are prone to severe overcrowding. These housing strategies are helped by the emergence of online mediated platforms that provide an accessible way to market and deliver housing by the room (Crommelin et al., 2018; Parkinson et al., 2019). Room sharing is the most extreme form of group households where multiple, unrelated individuals share a bedroom (Nasreen & Ruming, 2018). House and room sharing is concentrated in apartments, studios, and granny flats (Sarkar and Gurran 2017; cited in Nasreen & Ruming, 2018), housing types that are concentrated in the inner suburbs of Australia’s larger cities.^19^

**Table 4:** Proportion (%) of persons living in severely crowded dwellings by personal characteristics: Australia and selected state capital cities, 2016

	Australia	Sydney	Melbourne	Brisbane	Adelaide	Perth
Female^1^	46	43	41	47	43	47
Aged under 35 years^1^	74	74	77	77	74	78
Aged 35–64 years^1^	23	23	20	20	23	20
Aged 65 years and over^1^	3	3	2	3	2	2
Indigenous^1^	32	1	1	4	2	9
Attending tertiary institution (inc TAFE^)^1^	13	21	22	16	25	16
Country of birth: Australia^1^	51	24	25	36	22	31
Country of birth: Asia ^1,2^	35	58	54	33	58	49
Speaks English only^1^	25	18	20	41	19	31
Total personal income < $400/week^3^	54	47	53	51	66	52
Married (registered)^3^	32	34	31	25	34	31
Never married (registered)^3^	59	57	61	66	61	61
Divorced, widowed or separated^3^	9	8	8	9	6	8
Employed full-time^3^	18	22	20	23	14	21
Employed part-time^3^	19	26	24	22	17	23
Not in the labour force^3^	46	38	40	39	52	40
Batchelor degree or above^3,4^	14	22	19	15	14	15
Year 12 or below^3,4^	60	56	59	58	65	60

^1^All persons in severely crowded dwellings: The count is Australia = 51,092; Sydney = 15,115; Melbourne = 8037; Brisbane = 3136; Adelaide = 1587; Perth = 2011

^2^Asia: South-East Asia, North-East Asia, Southern and Central Asia regions (ABS Standard Australian Classification of Countries)

^3^Persons aged 15 years and above in severely crowded dwellings: The count is Australia = 39,050; Sydney = 12,765; Melbourne = 6474; Brisbane = 2465; Adelaide = 1270; Perth =1568

^4^Highest educational attainment, includes ‘Certificates I & II’ and ‘No educational attainment’

^TAFE: Tertiary and Further Education

Source: ABS Census TableBuilder: Counting Persons, Estimating Homelessness dataset, 2016

**Table 5:** Proportion (%) of persons staying temporarily with other households (couch surfers) by personal characteristics: Australia and selected state capital cities, 2016

	Australia	Sydney	Melbourne	Brisbane	Adelaide	Perth
Female^1^	41	41	44	40	37	37
Aged under 35 years^1^	46	53	54	45	52	45
Aged 35–64 years^1^	43	40	39	43	41	46
Aged 65 years and over^1^	11	7	7	12	8	8
Indigenous^1^	6	4	2	5	7	5
Attending tertiary institution (inc TAFE^)^1^	6	9	9	6	6	7
Country of birth: Australia^1^	72	59	63	71	76	64
Country of birth: Asia ^1,2^	8	16	14	7	7	9
Speaks English only^1^	83	67	70	85	84	84
Total personal income < $400/week^3^	44	39	40	44	50	45
Married (registered)^3^	21	15	17	22	13	17
Never married (registered)^3^	53	60	61	53	60	57
Divorced, widowed or separated^3^	25	25	22	26	26	26
Employed full-time^3^	23	29	27	23	16	22
Employed part-time^3^	13	16	14	13	13	13
Not in the labour force^3^	44	36	39	45	44	42
Batchelor degree or above^3,4^	15	23	21	16	12	16
Year 12 or below^3,4^	47	44	44	48	53	45

^1^All persons staying temporarily with other households: The count is Australia = 17,722; Sydney = 2751; Melbourne = 2113; Brisbane = 1773; Adelaide = 947; Perth = 1169

^2^Asia: South-East Asia, North-East Asia, Southern and Central Asia regions (ABS Standard Australian Classification of Countries)

^3^Persons aged 15 years and above staying temporarily with other households: The count is Australia = 16,155; Sydney = 2514; Melbourne = 1909; Brisbane = 1645; Adelaide = 870; Perth = 1096

^4^Highest educational attainment, includes ‘Certificates I & II’ and ‘No educational attainment’

^TAFE: Tertiary and Further Education

Source: ABS Census TableBuilder: Counting Persons, Estimating Homelessness dataset, 2016

In many ways, ‘couch surfers’ are a mirror image of the severely crowded homeless (see Table 5). The majority (54%) are 35 years or over with one in four (Australia-wide) the product of marital break-up. Australia-wide a large majority (72%) are Australian born and most (92% of the Australian born) from a non-indigenous background. Across the state capitals these personal characteristics are again typically homogenous, though the Australian born are less prominent among Sydney and Melbourne’s ‘couch surfers’. Older low-income age groups are less inclined to form group households, and being single, or the product of biographical disruption, found it increasingly difficult to sustain independent living arrangements as affordable stocks of rental housing declined over the study period. Couch surfing is then a more likely coping strategy, especially as they are not drawn from ethnic groups prone to form multigenerational households. It is a more viable plan if friends or relatives are willing and able to help, and this is more likely if the homeless are single, than families. It is also more likely if friends or relatives happen to live in housing with some spare space to accommodate an extra person. Couch surfers are, therefore, increasingly found on the urban fringe where more affordable housing is retreating (see Table 3) and larger properties with spare bedrooms are more common.^20^ Also important is an expected positive correlation between couch surfers’ low incomes and that of their support networks, an association more likely among residents in middle and outer suburbs of the capital cities.

## Conclusion

Our empirical analyses support four key findings. Firstly, regional rates of homelessness in Australia are becoming more uniformly distributed and this has been a persistent trend over the timeframe 2001–2016. Secondly, homelessness is becoming more urbanised. The capital cities share of national homelessness has surged, but capital cities share of the national population has been steady over the same timeframe. This urbanisation of homelessness is, therefore, unlikely to be due to rural and regional homeless persons moving into capital city locations *as part of general mobility patterns* among the national population.^21^ Thirdly, severe crowding has grown at a faster pace than any other type of homelessness to become the dominant form of homelessness. Capital cities have increased their share of severe crowding to account for about two-thirds of the 2016 total. Finally, there are important intra-city spatial dynamics. Rental housing affordable for low income households retreated to the middle and outer ring of capital city suburbs. Those vulnerable to homelessness among the young with very low household incomes, and residing relatively close to CBDs, responded by crowding into available housing in order to economise on housing costs. Meanwhile older, very low income persons, especially singles vulnerable to homelessness and unable to access boarding house or supported accommodation, responded by couch surfing typically in middle and outer suburbs where more spacious affordable rental housing retreated over the study period.

This new geography of homelessness is unlikely to be transient as the shrinking supply of affordable rental housing it is associated with is a long run trend. It also has implications for policy makers. From the perspective of homelessness policy our spatial analysis raises questions about the continued concentration of supported accommodation and boarding houses in central city locations. ‘Couch surfers’ are gravitating to outer suburban locations distant from these homelessness supports. An outreach service presence in outer suburban areas is then critical to assist in assessment and resettlement processes, and may comprise private rental brokerage, support to access social housing or permanent supportive housing where appropriate. These interventions can help prevent couch surfers moving into more chronic experiences of homelessness.

The surge in severe crowding homelessness appears to be the product of chronic shortages of affordable private rental housing in middle and inner ring suburbs. Younger recently arrived migrants, including international students, are most impacted and targeted housing support and regulatory reform (Louth & Huang, 2020) for these groups is required. Soaring rates of overcrowding homelessness also raises public health concerns. In the twentieth century severe crowding’s association with infection and disease prompted slum clearance. The recent COVID-19 pandemic serves as a timely reminder of these concerns. High rates of transmission in congregate homelessness accommodation options and high-rise social housing may prompt a rethink on the design of accommodation options targeted on the insecurely housed.

This paper adds to the emerging body of work documenting the spatial dynamics of homelessness in Australia, and provides a more geographically nuanced picture of the relationship between supplies of affordable rental housing and homelessness in Australia’s capital cities. Examining the spatial distribution of homelessness can help to guide the allocation of resources such as homelessness services and other interventions, as well as inform a policy area that has to date been notably aspatial. As our study has shown, the changing geography of affordable rental housing is especially important to understanding this policy area.

However, there are other possible drivers of the spatial dynamics of homelessness that future research might explore. These include changes in the types of rental housing stock that are supplied in an age when the use of internet platforms for intermediation are becoming more important. Finally, there is income inequality and its relationship to broader processes of urbanisation, densification and the socio-spatial polarisation in Australian cities.

## Footnotes

1 While the method of enumeration has remained the same, the 2016 Census shifted to being mainly online and this may have affected coverage of people experiencing homelessness.

2 For detailed information on the enumeration methodology for 2016 see: https://www.abs.gov.au/methodologies/census-population-and-housing-estimating-homelessness-methodology/2016

3 While data is available in the Specialist Homelessness Services Collection (SHSC) (AIHW, 2020), this data source does not adequately capture non-service users such as couch surfers or those living within severely crowded dwellings. Furthermore, data on service users is not available prior to 2011 and in later years is limited to areas where SHS are present, with few and sometimes no SHS in remote parts of Australia.

4 The RA classification divides Australia into five categories based on a measure of relative access (via road distances) to services (see ABS, 2018b). The RA classification is constructed using very fine spatial units and is not published at the SA3 level. Consequently, 163 comparatively large SA3s recorded more than one type of RA. In these SA3s, the RA type holding the highest proportion of the SA3s’ population was extended to the whole SA3. The ABS ‘SA3 2016 to RA 2016′ correspondence table provided the population information: (https://www.abs.gov.au/websitedbs/D3310114.nsf/home/Correspondences).

5 A similar definition of inner, middle and outer rings is employed in Gitelman and Otto (2012).

6 ‘Affordable’ is a rental amount that is less than 30% of the upper value of the household income quintile range. In 2016, households in the lowest 40% of the national income distribution had incomes of up to $1104/week and thus the corresponding affordable dwelling rent was up to $332/week. This approach is based on that used in the series of reports that culminated in Hulse et al. (2019) and was also used in Parkinson et al. (2019).

7 We also computed a Herfindahl (H) index. The H index never exceeds 0.01, suggesting that SA3s outside the largest regions have uniformly small shares of national homelessness. There is also a trend decline through 2001–2016.

8 An earlier version of this paper reported an index of dissimilarity for each capital city and Census dates 2001 through to 2016. It revealed declines in severe crowding segregation in all cities indicating that differences between the spatial distribution of severe crowding homelessness and other forms of homelessness are narrowing (results available from the authors on request).

9 Details on the distribution of people accommodated in homelessness services, boarding houses as well as rough sleepers are available from authors on request.

10 We are grateful to an anonymous referee for suggesting this idea.

11 A positive magnitude (negative) signals a surplus (deficit). See section 2 for details on construction of this measure.

12 Distance from CBD calculation is described in section 2. Population density is obtained by dividing the population of the SA3 by the area of the SA3 to give the number of persons per square kilometre.

13 Pearson correlation coefficients between the distance from CBD and population density measure in 2001 and 2016 are reported below:

**Table:**

	Adelaide	Brisbane	Perth	Melbourne	Sydney
2001	−0.657***	−0.445***	−0.627***	−0.843***	−0.792***
2016	−0.595***	−0.407***	−0.625***	−0.841***	−0.767***
*** p < 0.01, ** p < 0.05					

14 Coefficient estimates imply that shortages of affordable rental housing persist out to a radial distance of 26.2 kms from Sydney’s CBD.

15 In Sydney, for example, coefficient estimates imply that shortages of affordable rental housing stretch out beyond the built up area of Sydney to 80 kms from the CBD in 2016 as compared to 26.2 kms in 2001.

16 Some caution is warranted here because in these two cities the insignificance of the distance from CBD coefficients could be unreliable due to severe multicollinearity with the population density variable. Correlation between these variables is highest in Melbourne and Sydney (see footnote 14).

17 The indigenous are overrepresented in severe crowding homeless numbers Australia wide, but this is a feature that does not transfer to the state capitals, with the exception of Perth.

18 In 2011 only 18% of severe crowding homelessness were born in Asia. The share almost doubled to 35% in 2016, which correlates with strong growth of 38% in Asian international student tertiary enrolments, from 211,963 in 2011 to 291,860 in 2016 (https://stats.oecd.org/Index.aspx?DataSetCode=EDU_ENRL_MOBILE).

19 In Sydney, Nasreen and Ruming’s (2019, p156) study finds that ‘the highest concentration of shared rooms is found close to the CBD, with inner-city LGAs accounting for 40% of the listed shared rooms’. For group households in inner-ring suburbs, they estimate a severe crowding rate of 40%.

20 Goodman et al. (2010) find a modest increase in average floor space from 135m^2^ to 159m^2^ in middle ring Melbourne municipalities, while in the outer ‘growth’ municipalities there was a strong increase from 138m^2^ to 192m^2^. Meanwhile, median floor spaces in inner municipalities shrunk from 107m^2^ in 1990 to 81m^2^ in 2007.

21 Use of longitudinal data sets such as the Australian Journeys Home panel data set could shed light on these mobility patterns. Batterham (2020) finds that while people experiencing homelessness move frequently, their moves are not more likely to cross greater capital city or balance of state area boundaries than those of private renters.
